# From Discovery to Practice and Survivorship: Building a National Real‐World Data Learning Healthcare Framework for Military and Veteran Cancer Patients

**DOI:** 10.1002/cpt.1425

**Published:** 2019-04-29

**Authors:** Jerry S. H. Lee, Kathleen M. Darcy, Hai Hu, Yovanni Casablanca, Thomas P. Conrads, Clifton L. Dalgard, John B. Freymann, Sean E. Hanlon, Grant D. Huang, Leonid Kvecher, George L. Maxwell, Frank Meng, Joel T. Moncur, Clesson Turner, Justin M. Wells, Matthew D. Wilkerson, Kangmin Zhu, Rachel B. Ramoni, Craig D. Shriver

**Affiliations:** ^1^ Department of Medicine/Oncology Keck School of Medicine University of Southern California Los Angeles California USA; ^2^ Department of Chemical Engineering and Material Science Viterbi School of Engineering University of Southern California Los Angeles California USA; ^3^ Lawrence J. Ellison Institute for Transformative Medicine University of Southern California Los Angeles California USA; ^4^ Henry M. Jackson Foundation for the Advancement of Military Medicine Bethesda Maryland USA; ^5^ Center for Strategic Scientific Initiatives National Cancer Institute Bethesda Maryland USA; ^6^ Office of Research and Development Department of Veterans Affairs Washington DC USA; ^7^ Department of Obstetrics & Gynecology Gynecologic Cancer Center of Excellence Uniformed Services University of the Health Sciences and Walter Reed National Military Medical Center Bethesda Maryland USA; ^8^ John P. Murtha Cancer Center Research Program Department of Surgery Uniformed Services University of the Health Sciences and Walter Reed National Military Medical Center Bethesda Maryland USA; ^9^ Chan Soon‐Shiong Institute of Molecular Medicine at Windber Windber Pennsylvania USA; ^10^ Department of Obstetrics and Gynecology Inova Schar Cancer Institute Inova Fairfax Hospital Falls Church Virginia USA; ^11^ Department of Anatomy, Physiology, and Genetics Uniformed Services University of the Health Sciences Bethesda Maryland USA; ^12^ The American Genome Center Collaborative Health Initiative Research Program Uniformed Services University of the Health Sciences Bethesda Maryland USA; ^13^ Cancer Imaging Informatics Lab Leidos Biomedical Research, Inc. Frederick National Laboratory for Cancer Research Frederick Maryland USA; ^14^ Massachusetts Veterans Epidemiology Research and Information Center Veterans Affairs Boston Healthcare Boston Massachusetts USA; ^15^ Department of General Internal Medicine Boston University School of Medicine Boston Massachusetts USA; ^16^ Joint Pathology Center, National Capital Region Medical Directorate Defense Health Agency Silver Spring Maryland USA; ^17^ Department of Pediatrics Division of Genetics Walter Reed National Military Medical Center Bethesda Maryland USA; ^18^ Department of Pathology Walter Reed National Military Medical Center Bethesda Maryland USA; ^19^ Department of Surgery Uniformed Services University of the Health Sciences and Walter Reed National Military Medical Center Bethesda Maryland USA

The Applied Proteogenomics OrganizationaL Learning and Outcomes (APOLLO) network is implementing a prospective curation and translation of real‐world data (RWD) into real‐world evidence (RWE) within the learning healthcare environment of the Department of Defense and Department of Veterans Affairs. To support basic, translational, clinical, and epidemiological sciences, APOLLO will release data to public repositories for secondary analysis to assist others in assessing whether similar molecular‐driven clinical practice guidelines will improve health outcomes for their relevant cancer populations.

In the United States, > 80% of patients with cancer are initially diagnosed and treated in a community hospital setting rather than an academic hospital setting. Despite the increased adoption of electronic health records (EHRs), the lack of interoperable health information systems makes it challenging to aggregate RWD generated from a cancer patient’s journey before diagnosis, during treatment, and throughout survivorship. RWD might include data collected as part of routine health and cancer care delivery or for research (translational, implementation science, and/or epidemiological) efforts. Longitudinal collection of RWD is essential to generating RWE and is often absent when elucidating long‐term consequences of care strategies.

Recent studies have demonstrated the success of individualized cancer care strategies enabled by molecular profiling and targeted therapies. In the past 2 years, the US Food and Drug Administration (FDA) has approved tumor site–agnostic, biomarker‐driven cancer treatments and next‐generation sequencing *in vitro* diagnostic devices.^1^ A parallel review process by the Center for Medicare & Medicaid Services led to a national coverage determination next‐generation sequencing‐based *in vitro* diagnostics. The rapid development and approval of such technologies underscored this widening gap in capturing real‐world use of molecular‐driven cancer care to generate RWE to help inform regulatory and clinical decisions.^2^

Conducting valid real‐world studies requires data quality assurance through auditable data abstraction methods and incentives to drive electronic capture of data during delivery of care.^2^ The Department of Veterans Affairs (VA) has the nation's largest integrated healthcare system with over 9 million veterans enrolled and is a high‐volume provider of cancer care with nearly 50,000 incident cancer cases reported in 2010.^3^ The VA Office of Research and Development has as its three major priorities to: (i) enhance veteran access to multisite clinical trials, (ii) make VA data a national resource, and (iii) increase the real‐world impact of research findings. The VA Office of Research and Development's national Cooperative Studies Program^4^ and data resources enable researchers to access and identify initial cohorts for further studies to advance RWD analysis have been leveraged through partnerships with federal collaborators to further a learning health care system within the VA. The Department of Defense (DoD) Military Health System (MHS) is responsible for maintaining the health and readiness of 1.7 million active‐duty and reserve service members (SMs) and caring for 9.4 million beneficiaries in TRICARE health benefit plans. The John P. Murtha Cancer Center at Uniformed Services University and Walter Reed National Military Medical Center offers a comprehensive cancer care operational view in 64 capability areas to proactively mitigate and close gaps in cancer care and research in the MHS. The John P. Murtha Cancer Center utilizes agreements with other federal agencies and extramural collaborators to provide return on investment by deploying the most robust and modern molecular technologies under various programs. The administrative and medical care data from both direct and indirect care are stored in the military data repository, which includes detailed information on demographics, diagnoses, diagnostic procedures, prescriptions, ancillary and radiology services, treatments, cost of care, and vital status. The DoD also has a cancer registry that collects detailed data on cancer diagnosis and features, including some cancer biomarkers. These RWD have been widely used for cancer research among DoD beneficiaries.^5^, ^6^

Leveraging the two largest nationwide connected healthcare systems, the APOLLO network was launched in 2016 with the intent of curating longitudinal RWD and health outcome data to create and assess adoption of new molecular‐driven clinical practice guidelines. By developing, defining, and aligning RWD elements of MHS, patients with cancer from prediagnosis through survivorship among the federal and civilian partners, the APOLLO network is implementing an integrated multifederal network for prospective curation and translation of RWD into RWE in a learning healthcare environment that will assist other payers in assessing whether similar clinical practice guidelines will improve health outcomes for their relevant populations.

## MOVING TOWARD RWD: LESSONS LEARNED AND ONGOING PILOTS TO BUILD THE APOLLO ECOSYSTEM

Previous large‐scale tumor characterization projects, such as The Cancer Genome Atlas and the ongoing Clinical Proteomics Tumor Analysis Consortium, focused on analyzing the genomics and proteomics profile of tumors at a single time point.^7^ The lack of focus on longitudinal RWD collection limits the clinical utilization of these programs’ data.^8^ APOLLO is distinct from The Cancer Genome Atlas and other previous tumor characterization projects as it was focused on integrated proteogenomic analyses, the collection of longitudinal RWD, and development of a sustainable collection pipeline from its inception. The foundation of the approach is a network of biospecimen collection sites throughout the DoD and VA plus select civilian sites. APOLLO tissue collection is infused into pathology departments to preserve patient care, optimize collections, and control for preanalytic variables while involving the local organizations as true partners. This culture of collaboration also promotes the capture of longitudinal clinical, radiology imaging, and patient data throughout patients’ disease cycles that can otherwise be difficult to obtain. This culture expands to Clinical Laboratory Improvement Amendment (CLIA) laboratories, biobanking, imaging characterization, and proteogenomic analysis centers to form a robust APOLLO ecosystem that will be leveraged to enable additional longitudinal oncology studies of both established and new patients.

**Figure 1:**
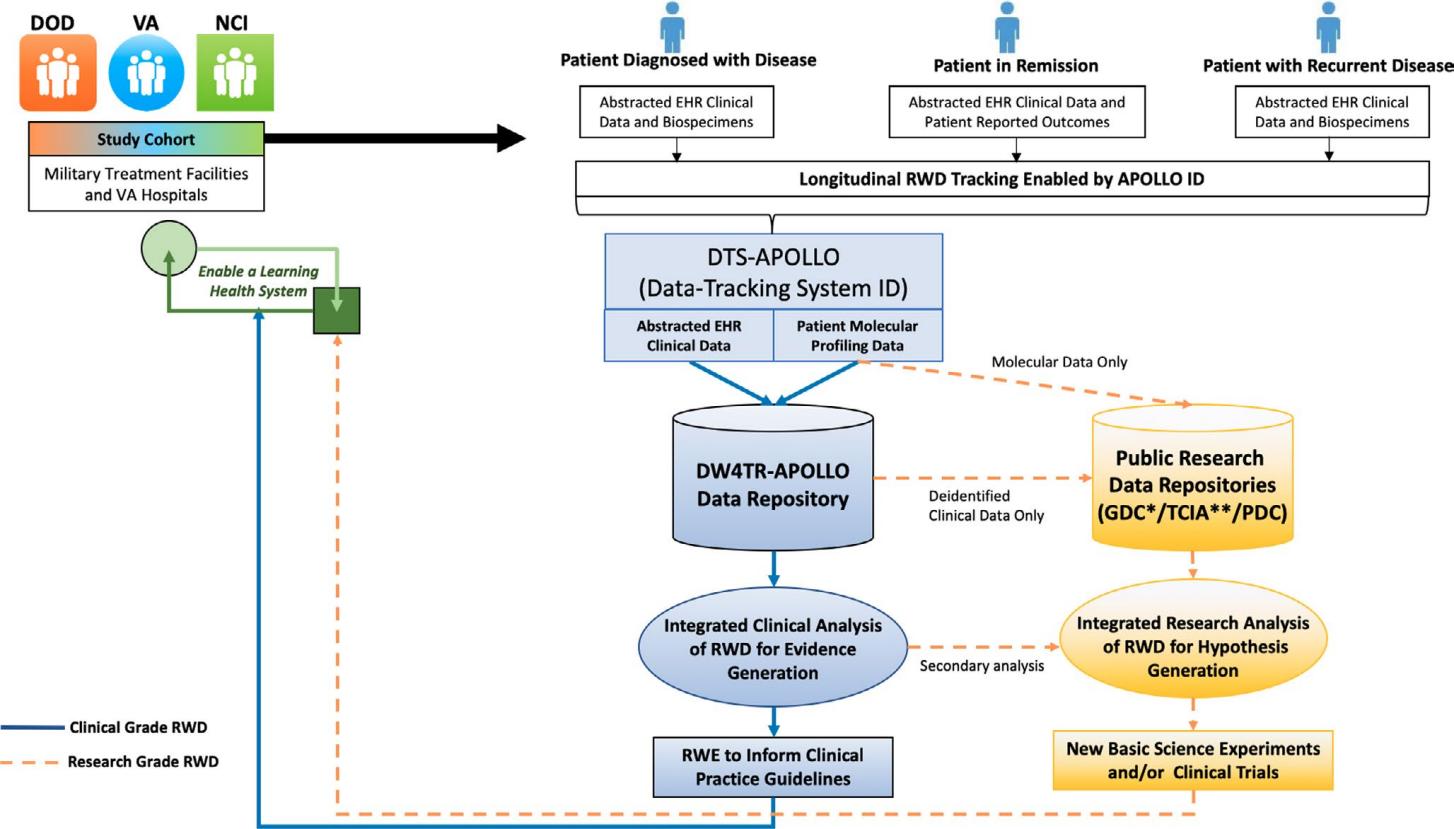
Applied Proteogenomics OrganizationaL Learning and Outcomes (APOLLO) data ecosystem and workflow to enable longitudinal real‐world data (RWD) collection and analysis. Clinical activities are separated from research functions by a firewall so that only de identified, limited datasets are available for research and further, only safe‐harbor datasets are made publicly available. Patient will be followed from the time of diagnosis through remission and when disease recurs, for as long as possible. Tracking of all such RWD is enabled by APOLLO IDs in a program‐wide Data Tracking System for APOLLO (DTS‐APOLLO). Activities in molecular center are tracked by local LIMS with metadata and higher‐level molecular data tracked in DTS‐APOLLO. Transactional data in DTS‐APOLLO will be quality assured and integrated in the Data Warehouse for Translational Research for APOLLO (DW4TR‐APOLLO) for integrated analysis to generate real‐world evidence (RWE), which will in turn directly impact patient clinical services. Lower‐level raw molecular and imaging data of very large size, on the other hand, will be directly uploaded to public data repositories, including The Cancer Imaging Archive (TCIA),^11^ Genomic Data Commons (GDC),^12^ and upcoming Proteomic Data Commons (PDC) maintained by the National Cancer Institute (NCI) following appropriate protocols and regulatory procedures coordinated through DW4TR‐APOLLO. Such raw data, after integration with the data in the DW4TR‐APOLLO enabled by APOLLO ID, will become substrates for integrated research analysis for hypothesis generation and testing, which will be the basis for the design of new scientific experiments and clinical trials with results will eventually impact future patient clinical care. Solid lines are for clinical‐grade RWD and dotted lines for research‐grade RWD. DoD, Department of Defense; EHR, electronic health record; VA, Veteran's Affairs.

To maximize longitudinal clinical data collection, APOLLO uniquely designed a combination of disease‐specific pilot retrospective studies of hundreds of cases (APOLLOs 1–4) and prospective studies of ~ 8,000 cases (APOLLO 5). Successes and lessons learned during the implementation of these pilot projects, as well as those from past large‐scale molecular and clinical studies, are being leveraged to successfully forge the APOLLO ecosystem. Central to generating RWE from RWD in combination with molecular data is the challenge of balancing effective biospecimen matching and integration of data from multiple modalities from the same patient while maintaining accuracy and privacy over time. One way the network tackled this issue was bringing together early stakeholders to develop and adopt a prospectively generated unique APOLLO participant and aliquot identifiers (APOLLO ID; **Figure** **1**). APOLLO ID will also be linked to a 128‐byte global unique participant and aliquot identifiers with an “AP‐” prefix when data are uploaded to public repositories for secondary analysis. The APOLLO system is electronically supported by an enterprise informatics infrastructure, which includes a Data Tracking System (DTS‐APOLLO) for transactional activities, a Data Warehouse for Translational Research for (DW4TR‐APOLLO),^9^ and a network of connected public data repositories to support capturing, management, and delivery of RWD to the study team and the public to enable discovery of RWE. Initial pilot datasets have been successfully uploaded to the National Cancer Institute's Genomic Data Commons and The Cancer Imaging Archive (TCIA) from both VA and DoD studies. The length of patient follow‐up time within APOLLO will be pre‐estimated for each cancer type using prior literature rather than by duration of a funding cycle, so advanced planning will enable continued capturing of such data from both the regulatory and technical perspectives.

## LOOKING AHEAD: INITIAL EFFORTS TO ELEVATE RWD TO RWE

The APOLLO program aspires to accelerate the application of next‐generation proteogenomic profiling with deep baseline and longitudinal RWD from DoD and VA EHRs and research records into RWE for FDA‐approved tests and treatments for development and deployment of tools and strategies used in the prevention, diagnosis, and treatment of cancer. These activities support readiness and health by empowering patients and providers to optimize their care and health through customized and enterprise solutions. The program will deploy both retrospective and prospective observational designs with provisions for clinical trial participation. Select civilian cohorts with aggressive or rare cancers will be incorporated with SMs and veterans to contribute diversity, events, experiences, and outcomes to the disease‐oriented and pan‐cancer cohorts to learn about, treat, and prevent cancers that develop in warfighters.

Types of clinical and research RWD that will be collected by the APOLLO network are listed in **Table** **1**. This program will require and utilize operationalized processes and procedures tracked via a user‐friendly APOLLO Dashboard. Integrated analyses will incorporate a deep complement of RWD from medical and research records. Sequencing and proteomic data generated by CLIA facilities and analytical core facilities will not only be analyzed using current clinical databases but will be available for iterative reanalysis over time applying new clinical databases and trusted sources to advance reinterpretation of the patients’ molecular profiling data to determine future access to new FDA‐approved drugs and/or clinical trial opportunities. This program will provide data in support studies of basic science, translational medicine, epidemiology, comparative effectiveness, cost‐effectiveness, and health disparities. Various data‐release provisions were incorporated into the APOLLO framework, including release to repositories for future research, clinical trials, indications and guidelines, dissemination to scientists, healthcare professionals, and the public, release to study doctors when research results meet guidelines for medical consideration for follow‐up and clinical assessments, and return to patients when the research results qualifies for release without clinical certification, as recommended recently by the National Academies of Sciences, Engineering, and Medicine.^10^

**Table 1:** **Types of RWD from medical and research records for APOLLO**

Captured into smart electronic clinical reporting and XML forms with data dictionaries, valid value requirements, logging features, and business rules. Data elements are labeled with a unique coded APOLLO ID participant identifier.
*Baseline data*: Registration, eligibility, consent, demographics, height, weight, risk factors, smoking status, marital status, type of insurance, medical history, medications, supplements, reproductive history, and family cancer history.
*Surgical treatment*: Surgical date, surgical procedures performed, AJCC stage with edition details, and disease site–specific surgical findings, including primary tumor size, disease distribution (location and size pre/post surgery), residual disease status, military disease, laterality, margins, redacted operative report(s), and comments.
*Pathologic findings*: Diagnosis date, definitive surgery date, ICD site and behavior codes, detailed College of American Pathology electronic cancer checklist^13^ with harmonized data dictionaries and conversion between versions, redacted pathology reports, including cytologic findings, clinical biomarker assessments, and other findings.
Case‐level data: Case organ type, lesion type, malignancy type, primary site of diagnosis, ICD‐10 code, histology code, TNM edition number, pathological group stage at diagnosis, CAP organ data creation status, and biomarker creation status.
Research pathology characterization: Baseline and in‐depth research pathology characterization will be provided and compared with the clinical diagnosis for tumor samples by expert pathologists and tissue imaging researchers. The types of annotation may include tissue composition details, clinical biomarker staining, and computer‐generated annotation in imaged slides with intact tumor tissues or tissues before and after laser microdissection.
*Molecular data*: Including redacted report, primary findings, and secondary findings when applicable from CLIA testing, clinical recommendations, clinical actions taken and outcomes, and XML data from CLIA assays when available implementing best practices and guidelines from the College of American Pathology, American Society of Clinical Oncology, National Comprehensive Cancer Network, and American College of Genetics and Genomic for risk assessments, interpretation, certification, and genetic counseling health conditions, including cancer.
DoD uses the Illumina TruSight Tumor 15 tumor profiling assay with plans to deploy the TruSight Oncology 500 tumor profiling DNA + RNA assay. VA uses the Personalis AC CancerPlus DNA + RNA assay to evaluate 181 clinically actionable genes or the PGDx Cancer Select 125 assay. Research analytical facilities generate next generation sequencing and multiple proteomic data. Immunoassay, cell‐free DNA, metabolomic, glycoprotein, and lipidomic data may be available in subsets.
*Clinical imaging*: May be acquired when accessible from medical records, imaging facilities, and research records with regulatory approval and consent at a baseline time point and as longitudinal series of collections to monitor and document disease distribution patterns and features utilizing enterprise solutions by the VA and customized solutions by DoD programs in partnership with TCIA.
Baseline details regarding imaging, including method, contrast, facility location, and dates for acquisition, curation, and submissions to and receipt of annotation.^11^
Disease‐oriented features will be annotated by expert radiologists using custom workstation configuration and standardized data dictionary, including assessments of mass: laterality, calcifications, thick septations, internal architecture; disease: presence, calcification, locations, shape; ascites or effusion: volume; lymphadenopathy: pathologic lymph nodes.
Computer‐generated features, including but not limited to segmentation using machine learning and artificial intelligence.
*Pharmacologic therapies*: Pharmacologic therapy status by regimen, treatment line, or indication with individual agent details with drug name, ICD‐O cancer site for treatment, doses, route/delivery method, cycles, date first dose/start date, date last dose/end date, dose schedule, active medication, dose reduction, treatment selection (approved assay or an integral, integrated, or exploratory biomarker), best response, and serious adverse events.
FDA indication with companion diagnostic assays: Non‐small cell lung cancer: Treat an EGFR exon 19 deletions or EGFR exon 21 L858R alterations with afatinib, gefitinib, or erlotinib; an EGFR exon 20 T790M alteration with osimertinib; ALK rearrangement with alectinib, crizotinib, or ceritinib; BRAF V600E with dabrafenib and trametinib. Melanoma: Treat BRAF V600E with dabrafenib or vemurafenib; BRAF V600E or V600K with trametinib or cobimetinib with vemurafenib. Breast cancer: Treat ERBB2/HER2 amplification with trastuzumab, ado‐trastuzumab emtansine, or pertuzumab. Colorectal cancer: Treat wild‐type KRAS (absence of mutations in codons 12 and 13) with cetuximab; wild‐type KRAS (absence of mutations in exons 2, 3, and 4) or wild‐type NRAS (absence of mutations in exons 2, 3, and 4) with panitumumab. Ovarian cancer: Treat BRCA1/2 alterations with rucaparib. Treatment of adult and pediatric patients with cancer with an NTRK fusion, including solid tumors and hematologic malignancies with larotrectinib.
*Radiotherapies*: Radiotherapy status by location, indication, radiation treatment line/regimen, laterality, field treated, radiation site code (ICD‐O), start date, end date, number of fractions, dose/fraction cGy, total dose cGy, best response, and best response assessment method, and comments.
*Outcome assessments*: If living: Disease status (alive with disease, no evidence of disease), date of last visit or date last activity if different than visit and capture individual dates of recurrence or progression with assessment method(s) and additional details when available. If deceased: Date of death and cause of death (cancer‐related, noncancer related, and unknown), if other cause then specify. Clinical trial participation will also be documented.
*Epidemiologic data*: May be provided directly by patients or with research staff during interviews with patients using a standardized data dictionary. Veterans may also contribute data through the Million's Veterans Program.
Patient demographics, including race, ethnicity, sex, marital status, education, employment, and military service. Medical history regarding health conditions, prior cancer diagnoses and treatments, height, and weight. Physical activity for 12 months prior to the current diagnosis. Alcohol history in entire life and currently. Tobacco products use in entire life and currently. Work environment, including occupations, exposures, and deployments. Family cancer history for blood relatives, including half blood relatives. Reproductive history for women.
*Patient‐reported outcomes*: Using validated instruments from trusted sources.
Patient Reported Outcomes Measurements for Personalizing Treatment (PROMPT Assessments): Quality of life using the 28‐item FACT‐G for physical, social/family, emotional, and functional well‐being. Global health using the 10‐item PROMIS Global Health version 1.2 instrument. Pain and fatigue using the 3‐item PROMIS Pain 3a and the 4‐item PROMIS Fatigue 4a instruments. Stress, anxiety, and depression combination using the 10‐item NIH ToolBox Perceived Stress, 4‐item PROMIS Anxiety 4a, and 4‐item PROMIS Depression 4a instruments. Symptoms using the 4‐item FACT‐NTX‐4, the 4‐item PROMIS Cognitive Function 4a, and the 4‐item PROMIS Sleep Disturbance 4a instruments. Support for daily living using the 11‐item PROMIS Instrumental Support version 2.0 instrument.
Focus assessments using validated instruments from trusted sources and working to deploy novel surveys to address gaps and support prevention, survivorship, palliative and end‐of‐life care to strengthen cancer capabilities across the continuum from prevention, early detection, treatment selection, mitigation of effects, rehabilitation, and survivorship, including palliative and end‐of‐life care. This may include assessments of barriers to care, patient preferences regarding treatment and care, resilience, cancer pain management, young adult survivorship, and serious adverse event reporting.

AJCC, American Joint Commission on Cancer; ALK, anaplastic lymphoma kinase; APOLLO, Applied Proteogenomics OrganizationaL Learning and Outcomes; BRAF, B‐type Raf; BRCA, breast cancer; CAP, College of American Pathologists; cGy, centigray; CLIA, Clinical Laboratory Improvement Amendment; DoD, Department of Defense; EGFR, epidermal growth factor receptor; ERBB, erythroblastic leukemia viral oncogene; FACT‐G, functional assessment of cancer therapy general; FDA, US Food and Drug Administration; HER2, human epidermal growth factor receptor 2; ICD‐10, International Classification of Disease‐10th edition; ICD‐O, International Classification of Disease for Oncology; KRAS, Kirsten RAt Sarcoma virus; NTRK, Neurotrophic tropomyosin receptor kinase; PGDx, Personal Genome Diagnostics; PROMIS, Patient‐Reported Outcomes Measurement Information System; RWD, real‐world data; TCIA, The Cancer Imaging Archive; TNM, Tumor, Node, Metastasis staging system; VA, Veteran's Affairs.

Translation of RWD into RWE is a key component of APOLLO with integrated systems for enhancing capabilities across the cancer care continuum, driving efficiencies, and enhancing quality, thereby improving health outcomes and the readiness of warfighters and the operational medical force. The full potential of APOLLO will be realized when interoperable EHRs are readily and securely exchangeable across the DoD and VA with enterprise solutions and clinical decision tools for molecular pathology, clinical imaging, patient‐reported outcomes, clinical trials, serious adverse events reporting, prevention clinics, rehabilitative and other supportive services, pain management, survivorship, palliative care, end‐of‐life care, research, and education.

## RETURN ON INVESTMENT: LEVERAGING RWD AND RWE FOR DOD, VA, AND THE GLOBAL CANCER ECOSYSTEM

Improvements in readiness, health care, and outcomes for SMs, veterans, health beneficiaries, and civilians will be achieved not only from deliverables generated by the APOLLO network but also from release of RWD and RWE to the public for secondary research. APOLLO patients may also benefit from release of research data that qualify either for clinical certification or direct release based on criteria, such as level and quality of the evidence. Federal agencies may also benefit from the generated agreements, established working groups, and taskforces with representation from the stakeholders and invited nonfederal experts, aligned resources and assets, integrated and expanded infrastructure and workforces, and the capabilities developed for APOLLO and operationalized across the DoD and VA for implementing precision oncology solutions to acquire and translate RWD from APOLLO into RWE for SMs, veterans, and the global cancer ecosystem.

## Funding

Funding for these efforts was provided from Uniformed Services University of the Health Sciences (USUHS) awards from the Defense Health Program to the Murtha Cancer Center Research Program (HU0001‐16‐2‐0014, C.D. Shriver and J.S.H. Lee), the Gynecologic Cancer Center of Excellence (HU0001‐16‐2‐0006, Y. Casablanca and G. Larry Maxwell), and HU0001‐16‐2‐004 (L. Kvecher and H. Hu) administered by the Henry M. Jackson Foundation for the Advancement of Military Medicine. This project has also been funded in whole or in part with federal funds from the National Cancer Institute, National Institutes of Health, under Contract No. HHSN261200800001E (J.B. Freymann).

## Conflict of Interest

The authors declared no competing interests for this work.

## Disclaimer

The contents of this publication are the sole responsibility of the authors and do not necessarily reflect the views, opinions, or policies of the USUHS, the Henry M. Jackson Foundation for the Advancement of Military Medicine, Inc., the Department of Defense (DoD), the Departments of the Army, Navy, or Air Force, Department of Health and Human Services, or Department of Veterans Affairs. Mention of trade names, commercial products, or organization does not imply endorsement by the U.S. Government.
